# Perioperative risk factors predict one-year mortality in patients with acute type-A aortic dissection

**DOI:** 10.1186/s13019-020-01296-8

**Published:** 2020-09-11

**Authors:** Yanwei Yang, Jiayi Xue, Huixian Li, Jiaqi Tong, Mu Jin

**Affiliations:** 1 Department of Anesthesiology, Beijing Friendship Hospital, Capital Medical University, Beijing, 100050 China; 2 Department of Anesthesiology, Beijing Anzhen Hospital, Capital Medical University, Beijing Institute of Heart Lung and Blood Vessel Diseases, and Beijing Engineering Research Center of Vascular Prostheses, Beijing, 100029 China; 3 Department of Anesthesiology, Guangzhou Women and Children’s Medical Center, Guangzhou, China; 4 Department of Anesthesiology, The First Hospital of Tsinghua University, Beijing, China

**Keywords:** Acute type a aortic dissection, Short-term mortality, Perioperative risk factor

## Abstract

**Objective:**

The goal of this study was to analyze perioperative risk factors to predict one- year mortality after operation for acute type A aortic dissection (AAD).

**Methods:**

A total of 121 consecutive patients undergoing Stanford type A AAD surgery in Beijing Anzhen Hospital were enrolled. Preoperative clinical and laboratory data from patients were collected.

**Results:**

Multivariable Cox regression analysis showed that significant factors associated with increased one-year mortality were elder age (year) (hazard ratio (HR) 1.0985; 95% confidence interval (CI) 1.0334–1.1677), intraoperative blood transfusion ≥2000 mL (HR 8.8081; 95% CI 2.3319–33.2709), a higher level of serum creatinine (μmol/L) at postoperative one day (HR 1.0122; 95% CI 1.0035–1.0190) and oxygenation index (OI) < 200 (mmHg) at the end of surgery (HR 5.7575; 95% CI 1.1695–28.3458).

**Conclusion:**

In this study, perioperative risk factors to predict one-year prognosis are age, intraoperative blood transfusion ≥2000 mL, postoperative OI < 200 mmHg and level of postoperative serum creatinine. The results aid in the comprehension of surgical outcomes and assist in the optimization of treatment strategies for those with perioperative risk factors to decrease one-year mortality.

## Background

Acute type-A aortic dissection (ATAAD) is a deadly cardiovascular event, and emergency surgery is typically indicated but associated with a 5–25% mortality rate [1–3]. Several predictors of short-term adverse events in acute aortic dissection have been investigated, including age [4], female predispositions [5], prolonged mechanical ventilation [6], hypotension [7], and impaired renal function [8]. Our previous study showed that postoperative mortality was less than one year (6). Thus this investigation provides insights into the one-year mortality rate following an operation for ATAAD to analyze the perioperative risk factors that predict short-term mortality.

## Materials and methods

The data used in this study were acquired from a previous clinical trial (ClinicalTrials.gov number: NCT01894334) [9], and the procedures were approved by the Beijing Anzhen Hospital Clinical Research Ethics Committee. Informed consent was obtained before surgery.

### Patients

All patients with ATAAD were enrolled in Beijing Anzhen Hospital between January 2013 to November 2014. ATAAD was defined as patients appearing with aortic dissection within two weeks of the onset of symptoms [10]. This study included patients aged 18 to 75 years diagnosed with ATAAD confirmed by computed tomography (CT) assisted angiography of aorta. Patients with severe coronary heart disease, severe cardiac tamponade, severe nervous system abnormalities, grossly incomplete medical record and patients died within 48 h after surgery were excluded. A total of 121 patients (93 men, 28 women) with a mean age of 46.6 ± 10.4 years were included in the final analysis.

### Surgical technique

The surgical technique has been described in detail previously [6]. In brief, after cardiopulmonary bypass (CPB) was established, the surgical procedure involved the deployment of a frozen elephant trunk, Cronus (MicroPort Medical, Shanghai, China), into the descending aorta, followed by total arch replacement with a 4-branched vascular graft (Maquet Cardiovascular, Wayne, NJ). Aortic valve or root procedures and concomitant surgeries were performed during the cooling phase. Allogeneic red blood cells were transfused to maintain a post-CPB hemoglobin level > 7 g/L and to correct coagulopathy after normalizing the thromboelastogram with fresh frozen plasma and platelets.

### Date collection

The patient’s profiles, such as age, gender, body mass index (BMI), smoking history (age commenced, age ceased, and average cigarettes smoked per day), hypertension history (age of onset and treatment), and diabetes history (age of onset and treatment), aortic diameter, left ventricular ejection fraction (LVEF), left ventricular end-diastolic dimension (LVEDd), were gathered from the case database. Perioperative clinical data from patients were also collected. Prolonged mechanical ventilation was defined as mechanical ventilation for more than 48 h after surgery.

### Statistical analysis

This article is a reanalysis of data published previously [6, 11]. Data analysis was performed using SPSS for windows, Version 18.0 (IBM, Armonk, NY, USA). Data was presented as mean ± standard deviation (or median [interquartile range]) and groups were compared using a Student’s *t*-test for normal distribution. Numbers (percentage) were compared using a Pearson chi-square or Fisher exact test. A Mann–Whitney U-test was performed for non-normal or skewed distributions. Cox proportional hazards models were used to compare the crude group with the adjusted group. Survival was assessed using the Kaplan–Meier method. All tests were two-sided, and statistical significance was set at *p* < 0.05.

#### Study endpoint

The primary endpoint of this study was the risk factors for one-year mortality in patients with Acute Type-A Aortic Dissection.

## Results

**Fig. 1:**
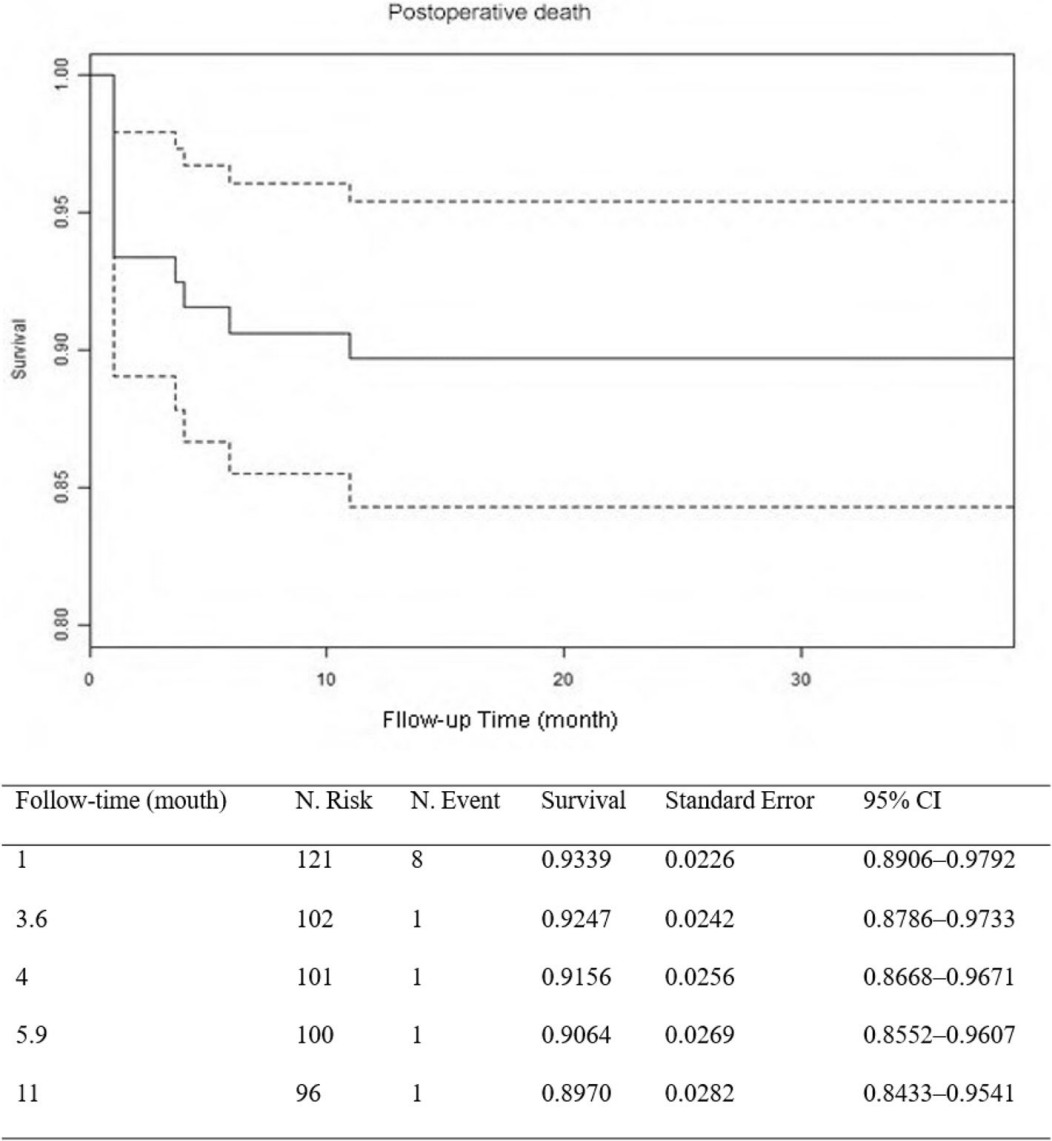
Kaplan-Meier survival in patients with Acute type-A aortic dissection (ATAAD) repair

The flow diagram was present in Fig. S1. Total arch replacement and frozen elephant trunk implantation (FET) was successful in all 121 patients and a modified Bentall procedure was performed in 66 patients with severe aortic regurgitation. The perioperative clinical profiles of all 121 patients were shown in Table 1. The in-hospital mortality rate was 6.6% (8/121) and overall postoperative one-year mortality was 9.9% (12/121). The patient life curves were shown in Fig. 1.

**Table 1:** Perioperative Clinical Profiles

Variables	Total	Alive	Death	HR	95% CI	*p* Value
Preoperative						
Number (*n*)	121	109	12			–
Age (year)	46.6 ± 10.4	45.7 ± 9.9	55.2 ± 11.1	1.0986	1.0318–1.1697	0.0033
Males, *n* (%)	93 (77)	84 (77)	9 (75)	0.8957	0.2425–3.3087	0.8688
BMI (kg/M^2^)	25.9 ± 3.2	25.9 ± 3.3	25.3 ± 2.7	0.9375	0.7820–1.1239	0.4852
History of smoking, *n* (%)	63 (52)	57 (52)	6 (50)	0.9001	0.2903–2.7912	0.8554
History of hypertension, *n* (%)	86 (71)	77 (71)	9 (75)	1.2185	0.3299–4.5012	0.7669
Time from onset of symptoms to surgery (d)	2.00 (1.00–5.00)	2.00 (1.00–5.00)	1.00 (0.95–2.25)	0.7173	0.4982–1.0328	0.0740
Aortic diameter (mm)	47.6 ± 8.2	47.2 ± 7.9	50.7 ± 10.0	1.0416	0.9816–1.1053	0.1785
Hear rate (beats/min)	77 ± 15	76 ± 15	78 ± 15	1.0103	0.9745–1.0474	0.5779
SBP (mm Hg)	114 ± 18	113 ± 18	115 ± 19	1.0044	0.9736–1.0362	0.7822
DBP (mm Hg)	57 ± 12	56 ± 12	59 ± 16	1.0277	0.9806–1.0771	0.2541
LVEF (%)	63 ± 9	62 ± 9	64 ± 6	1.0236	0.9415–1.1130	0.5843
LVEDd (mm)	51 ± 8	52 ± 8	49 ± 7	0.9412	0.8560–1.0349	0.2106
HB (g/L)	12.7 ± 1.4	12.8 ± 1.4	12.2 ± 0.9	0.7723	0.5246–1.1369	0.1903
PLC (10^9^/L)	175 ± 69	180 ± 68	132 ± 52	0.9897	0.9796–0.9998	0.0455
WBC (10^9^/L)	9.5 ± 3.5	9.5 ± 3.5	9.3 ± 2.6	0.9909	0.8374–1.1726	0.9156
LAC (mmol/L)	1.00 (0.80–1.30)	0.90 (0.80–1.30)	1.55 (1.00–2.17)	1.4802	1.1239–1.9493	0.0052
EuroSCOREII	5.00 (5.00–6.00)	5.00 (5.00–5.00)	5.50 (5.00–6.25)	1.8392	1.0416–3.2476	0.0357
Intraoperative						
B + S *n* (%)	66 (54.55%)	61 (55.96%)	5 (41.67%)	0.5943	0.1886–1.8726	0.3742
A + S *n* (%)	55 (45.45%)	48 (44.04%)	7 (58.33%)	1.6826	0.5340–5.3017	0.3742
Duration of surgery (min)	451 ± 102	443 ± 97	520 ± 133	1.0062	1.0013–1.0111	0.0126
Duration of CPB (min)	198 ± 53	196 ± 51	213 ± 69	1.0045	0.9959–1.0131	0.3045
Cross clamp time (min)	115 ± 38	114 ± 39	120 ± 32	1.0034	0.9900–1.017	0.6212
Lowest rectal temperature (°C)	25.8 ± 2.1	25.8 ± 2.1	25.5 ± 2.2	0.9176	0.6861–1.2272	0.5622
Allogeneic Red blood cells (units)	2.00 (0.00–4.00)	2.00 (0.00–4.00)	4.00 (1.50–6.25)	1.2395	1.0465–1.4682	0.0129
Blood transfusion > 2000 mL	18 (14.88)	13 (11.93)	5 (41.67)	4.8038	1.5229–15.1534	0.0074
End of surgery						
Heart rate (beats/min)	95 ± 17	94 ± 16	105 ± 17	1.0315	1.0065–1.0572	0.0132
SBP (mmHg)	117 ± 15	118 ± 14	114 ± 17	0.9805	0.9420–1.0206	0.335
DBP (mmHg)	62 ± 11	62 ± 10	61 ± 15	0.9917	1.0084–1.0481	0.7680
HB (g/L)	10.1 ± 1.9	10.1 ± 1.8	10.5 ± 2.5	1.0806	0.8150–1.4327	0.5901
PLC (10^9^/L)	109 ± 52	112 ± 54	84 ± 29	0.9879	0.9743–1.0017	0.0855
WBC (10^9^/L)	11.2 ± 4.4	11.3 ± 4.4	10.0 ± 3.5	0.9330	0.8005–1.0874	0.3748
LAC (mmol/L)	3.65 (2.00–5.75)	3.50 (1.90–5.45)	5.80 (4.85–8.90)	1.2738	1.1005–1.4744	0.0012
OI < 200 mmHg, *n* (%)	64 (52.89)	54 (49.5)	10 (83.3)	4.5814	1.0037–20.9106	0.0494
Postoperative						
Postoperative LVEF (%)	61 ± 8	61 ± 7	54 ± 14	0.9049	0.8421–0.9723	0.0064
Serum creatinine (μmol/L)	109 ± 52	104 ± 44	163 ± 80	1.0146	1.0067–1.0225	0.0003
Neurological deficits, *n* (%)	12 (9.92%)	9 (8.26%)	3 (25.00%)	3.4885	0.9436–12.8974	0.0611
prolonged mechanical ventilation *n* (%)	35 (28.93%)	27 (24.77%)	8 (66.67%)	5.6310	1.6932–18.7263	0.0048
Reexploration for bleeding or debridement, *n* (%)	11 (9.09%)	9 (8.26%)	2 (16.67%)	1.0932	0.1410–8.4735	0.9321
Tracheotomy *n* (%)	10 (8.26%)	7 (6.42%)	3 (25.00%)	4.4911	1.2135–16.6210	0.0245
CRRT *n* (%)	5 (4.13%)	3 (2.75%)	2 (16.67%)	5.8043	1.2696–26.5370	0.0233

*Abbreviations*: *A + S* ascending aorta replacement + Sun’s procedure, *B + S* Bentall + Sun’s procedure, *BMI* body mass index, *CI* confidence interval, *CPB* cardiopulmonary bypass, *CRRT* continuous renal replacement therapy, *DBP* diastolic blood pressure, *HB* hemoglobin, *HR* hazard ratio, *LAC* lactate, *LVEDd* left ventricular end-diastolic dimension, *LVEF* left ventricular ejection fraction, *OI* oxygenation index, *PLC* preoperative platelet count, *SBP* systolic blood pressure, *WBC* white blood cells

As shown in Table 1, the following variables differed significantly between the alive and death groups: years of age (Age, *p* = 0.0033), preoperative platelet count (PLC, *p* = 0.0455), preoperative serum lactate (LAC, *p* = 0.0052), EuroSCOREII (*p* = 0.0357), duration of surgery (*p* = 0.0126), intraoperative transfusion of blood products ≥2000 mL (*p* = 0.0074), postoperative heart rate (*p* = 0.0132), postoperative serum lactate (*p* = 0.0012), oxygenation index at end of surgery < 200 mmHg (OI, *p* = 0.0494), postoperative LVEF (*p* = 0.0064), postoperative serum creatinine (*p* = 0.0003), tracheotomy (*p* = 0.0245), continuous renal replacement therapy (*p* = 0.0233) and prolonged mechanical ventilation (*p* = 0.0048).

**Fig. 2:**
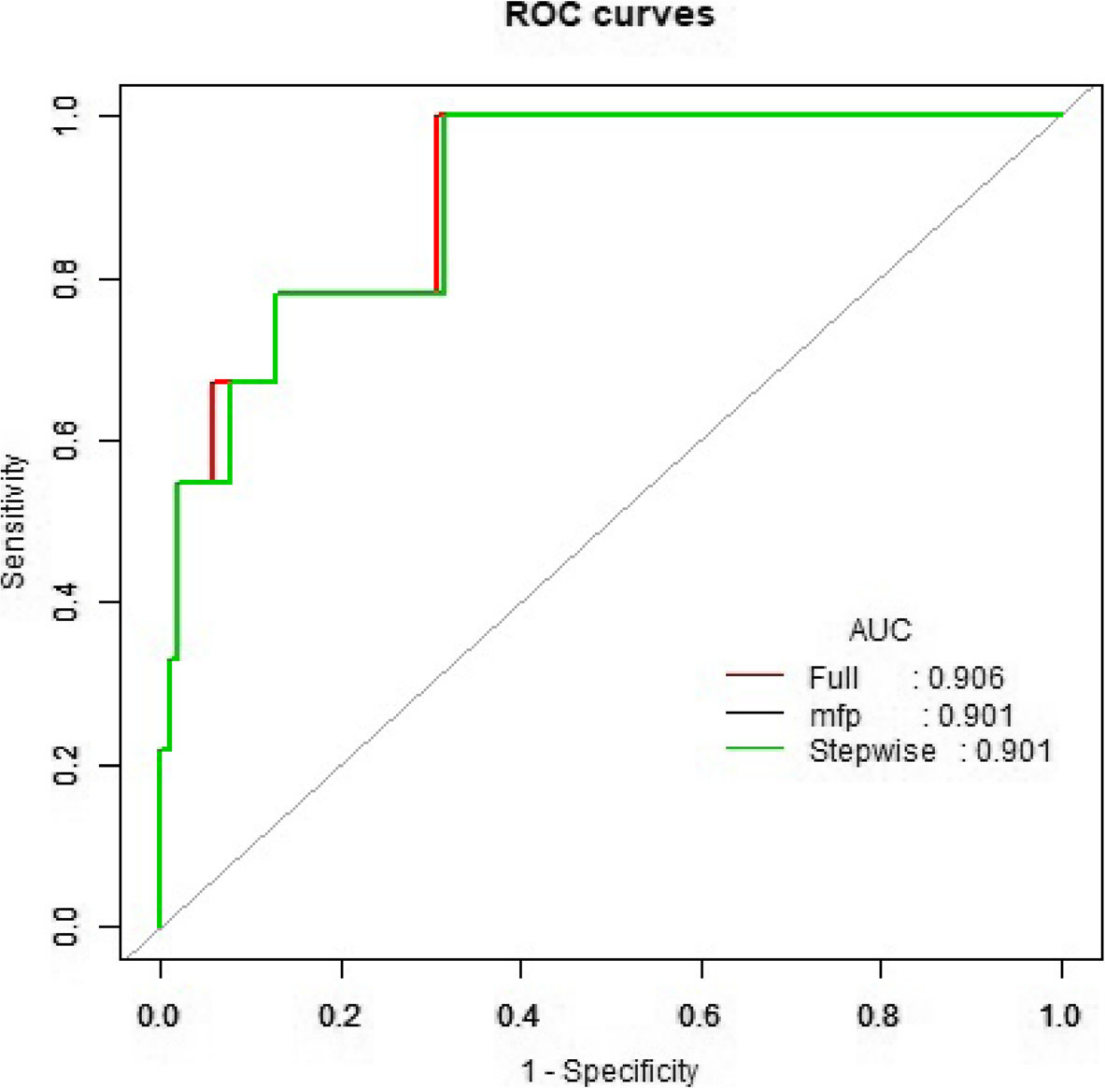
Receiver-operator characteristic (ROC) curve for the multivariable predictive model of one-year mortality in patients with AAD. Area under the ROC curve (AUC) (*P* value) was 0.906 (*P* < 0.001). *Full: Full model from observed data; MFP: Multiple fractional polynomial models from observed data; Stepwise: Stepwise selected model from observed data; p < 0.0001. AUC, area under curve; ROC, receiveroperating characteristic*

**Fig. 3:**
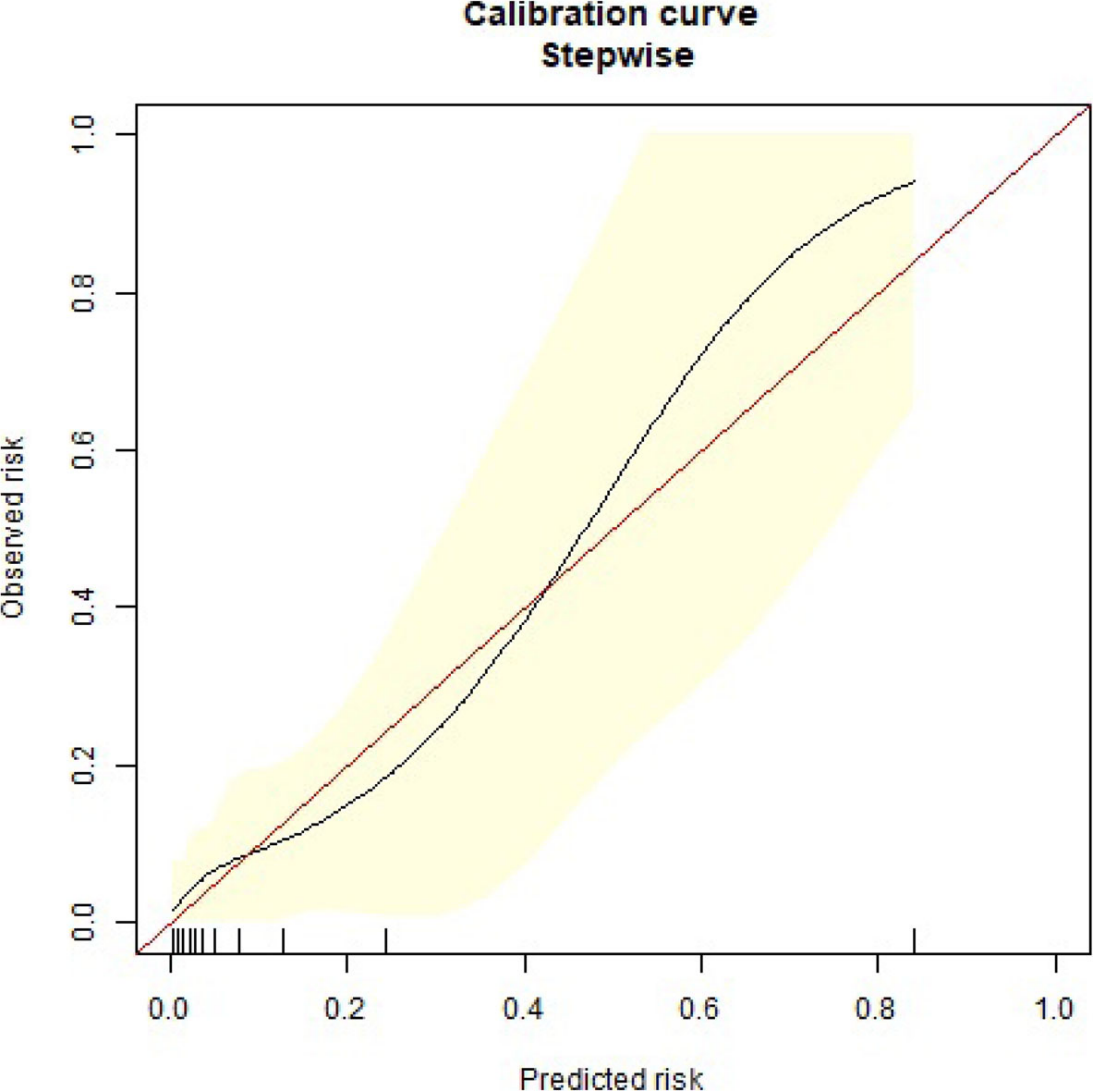
Calibration curve showing the predicted mortality risk against the observed one-year mortality risk in the overall cohort. Among patients with actual mortality of less than 40%, the model overestimated mortality risk by 10% or greater

Elder patients (hazard ratio (HR) 1.0999; 95% confidence interval (CI) 1.0334–1.1677; *p* = 0.0026), or those that underwent intraoperative blood transfusion ≥2000 mL (HR 8.8081; 95% CI 2.3319–33.2709; *p* = 0.0013), OI at the end of surgery < 200 mmHg (HR 5.7575; 95% CI 1.1695–28.3458; *p* = 0.0314) or had a higher level of serum creatinine at postoperative one day (μmol/L, HR 1.0112; 95% CI 1.0035–1.0190; *p* = 0.0043) had a higher risk of one-year mortality following surgery per Cox regression analysis (Table 2). The area under the receiver operating characteristic (ROC) curve was 0.906 (*p* < 0.001, Fig. 2), suggesting modest predictability. However, among patients with actual mortality of less than 40%, the model overestimated mortality risk by 10% or greater (Fig. 3).

**Table 2:** Risk Factors for One-year Mortality after Repair of Acute Type A Aortic Dissection

Variables	Crude			Adjusted^*^		
	HR	95% CI	*p* Value	HR	95% CI	*p* Value
Age (year)	1.0985	1.0334–1.1677	0.0026	1.0985	1.0334–1.1677	0.0026
Intraoperative Blood transfusion > 2000 mL	8.8081	2.3319–33.2709	0.0013	8.8081	2.3319–33.2709	0.0013
Serum creatinine (μmol/L) at postoperative one day	1.0112	1.0035–1.0190	0.0043	1.0112	1.0035–1.0190	0.0043
OI < 200 (mmHg) at the end of suegery	5.7575	1.1695–28.3458	0.0314	5.7575	1.1695–28.3458	0.0314

Abbreviations: *CI* confidence interval, *HR* hazard ratio, *OI* oxygenation index;

^*^ Adjusted for time from symptom onset to surgery, duration of surgery, delayed extubation, preoperative LAC, postoperative neurological complications, preoperative platelet count and EuroSCOREII

## Discussion

ATAAD is a rapidly progressing catastrophic disease associated with high morbidity and mortality. In this single-center clinical trial, the one-year mortality was 9.9%. This finding was consistent with the mortality presented in previous reports [12].

Marfan syndrome was the causative and exclusively factored in younger patients. In contrast, arterial hypertension and atherosclerosis were the causes in elderly patients [13, 14]. Affected by long-term hypertension and progressive atherosclerosis, the elderly and young people have different pathological and pathogenic mechanisms in the development of acute aortic dissection [15, 16]. In the present investigation, we also found that as age increases, so does the mortality rate. Although our study population is relatively young compared to previous studies, we still think the results may be related to the difference in the aforementioned pathogenic factors (such as hypertension and atherosclerosis) in recent years. In addition to hypertension and underlying connective tissue disorders, systemic pathological changes also result in end-organ damage such as chronic renal dysfunction. Importantly, each of these factors increases the risk of anesthesia, surgery, and postoperative mortality.

Serum creatinine is an important indicator of renal function. In our study, postoperative hyperphosphatemia creatinine suggests the possibility of postoperative renal injury. Patients with postoperative renal injury have more extended hospital stays, longer postoperative ventilator support time, and even increased mortality [17, 18]. The extension of the aortic dissection may involve renal ischemia or renal infarction on one or both sides of the renal artery, ultimately leading to renal insufficiency or kidney failure [19, 20]. Non-fluctuating perfusions and activation of inflammatory reactions during the extracorporeal circulation also lead to postoperative renal injury [21, 22].

Recent studies have shown that preoperative hypoxemia (HO) is an independent risk factor for acute lung injury (ALI) in patients with ATAAD [6]. Studies have shown that postoperative ALI will Prolonged mechanical ventilation, ICU and hospital stay [23]. It has also been reported that 5.3 to 16.7% of patients with ATAAD die from multiple organ dysfunction characterized by acute respiratory failure [24]. Postoperative patients are more prone to acute respiratory distress syndrome, decreased oxygenation index, increased postoperative ventilator use time, and increased ventilator-related pulmonary complications, which may be one of the reasons for the high short-term mortality rate [6].

AAD is characterized by the rapid development of an intimal flap separating the true and false lumen, blood flow through the non-endothelialized false lumen, tissue damage, and turbulence, each of which triggers coagulation. During surgery, CPB-induced coagulopathy, platelet activation and dysfunction and promoted coagulation factor consumption and excessive fibrinolysis [25, 26]. Therefore, patients undergoing surgery for AAD bleed excessively and require blood products and transfusions, which is the primary cause of surgical- mortality [27]. In our study, a large number of blood transfusions (≥2000 ml) during surgery is also a risk factor for postoperative one-year mortality.

### Study limitations

This study had three limitations. First, this trial was a retrospective analysis of prospectively collected data, and all inherent biases of retrospective analysis are inevitable. Second, all of the patients in our study were suitable for emergency AAD surgery without severe malperfusion and unstable hemodynamics and also excluded two patients who died within 48 h due to surgical procedure. The results of our cohort study might be more reflective of our clinical experience. Third, among some individuals with actual mortality of less than 40%, the model might overestimated mortality risk by 10%. Therefore, the use of the model may lead some patients to inappropriately overestimated mortality risk.

## Conclusions

In the study of ATAAD, there were several factors associated with higher one-year mortality, including elder age, intraoperative blood transfusion ≥2000 mL, postoperative OI < 200 mmHg and a higher level of postoperative serum creatinine. The results of our study aid in the comprehension of surgical outcomes and assist in the optimization of treatment strategies for those with perioperative risk factors to decrease short-term mortality.

## Supplementary information

**Additional file 1.** Figure S1. Flow diagram

## Data Availability

The dataset used and analyzed during the current study is available from the corresponding author upon reasonable request.

## Abbreviations

Abbreviated: Full Name
AAD: Acute Aortic Dissection
ATAAD: Acute type-A aortic dissection
CT: Computed Tomography
CPB: CardiopulmonaryBypass
BMI: BodyMass Index
LVEF: Left Ventricular Ejection Fraction
LVEDd: Left Ventricular End-diastolic Dimension
FET: Frozen Elephant Trunk
A + S: Ascending Aorta Aeplacement + Sun’s Procedure
B + S: Bentall + Sun’s Procedure
CI: Confidence Interval
CRRT: Continuous Renal Replacement Therapy
DBP: Diastolic Blood Pressure
SBP: Systolic Blood Pressure
HB: Hemoglobin
LAC: Lactate
OI: Oxygenation Index
PLC: Preoperative Platelet Coun
WBC: White Blood Cells
HO: Preoperative Hypoxemia
ALI: Acute Lung Injury
