# Supplementary figures and images for: Perioperative risk factors predict one-year mortality in patients with acute type-A aortic dissection
Source: J Cardiothorac Surg. 2020 Sep 11;15:249. doi: 10.1186/s13019-020-01296-8 (PMC7488853; doi:10.1186/s13019-020-01296-8)

Figure S1. Flow diagram.


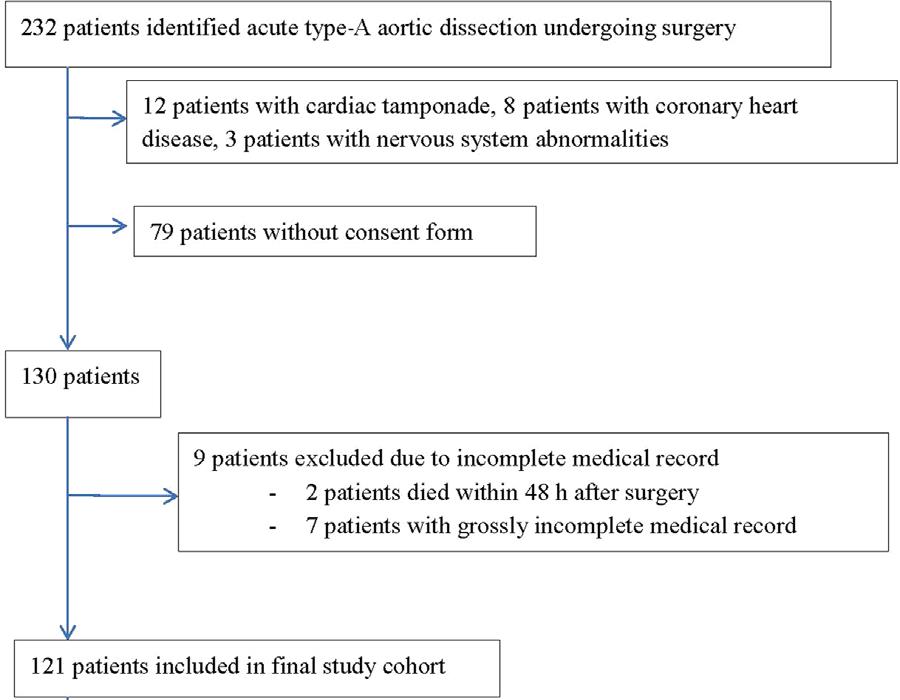

Supplement: Supplementary file 1 — Additional file 1. Figure S1. Flow diagram [file 13019_2020_1296_MOESM1_ESM.docx]
